# Cognitive function assessment should be included in preoperative evaluation

**DOI:** 10.7555/JBR.32.20180008

**Published:** 2018-04-15

**Authors:** David Li, Hong Liu

**Affiliations:** Department of Anesthesiology and Pain Medicine, University of California Davis Health, Sacramento, CA 95817, USA.; Department of Anesthesiology and Pain Medicine, University of California Davis Health, Sacramento, CA 95817, USA.

As the aging population continues to increase worldwide, a proportionally increasing number of elderly patients will be undergoing surgical procedures requiring anesthesia care. It has been estimated that approximately one of every three surgical procedures are performed on a patient who is 65 years of age or older^[1]^. The incidence of postoperative cognitive dysfunction (POCD) amongst the elderly population can vary significantly depending on patient’s preexisting cognitive function, the nature of the surgical intervention, and other associated risk factors. An 11.7% incidence has been reported of POCD three months following non-cardiac surgery^[2]^, which may be as high as 60% in cardiac surgery patients^[3]^. Despite the wide range of incidence being reported in the literature, it is apparent that POCD does occur on a regular basis. Such change in cognitive function which is characterized by problems in thinking and perception in surgical patients, regardless of how transient it is, can result in a significant clinical and financial impact on our health care system. Therefore, it is imperative for perioperative physicians to come up with an effective and yet reliable cognitive function assessment tool to help identify those at higher risk of POCD in order to better tailor their perioperative care.

## POCD clinical and financial impacts

POCD is a frequent complication after major surgery and contributes to increased mortality, prolonged duration of ventilation, longer length of stay in the intensive care unit (ICU) and hospital and higher treatment costs^[4]^. In recent years, greater attention has been paid to the negative impact of delirium on the health system and postoperative outcomes due to the increasingly budget conscientious health care practice. It has been estimated that delirium’s impact on patients during surgery or hospitalization results in more than $164 billion in US healthcare costs per year^[5]^. It is a huge burden on healthcare costs.

## How to screen for and diagnose POCD?

It is challenging to screen for and diagnose POCD in the perioperative setting. For instance, the diagnosis has mainly relied on administering various neuropsychological tests to patients to observe any decline in cognitive function. However, there is no consensus regarding which of the tests is the best^[3]^. Additionally, POCD is the result of a complex interplay of predisposing risk factors and precipitating perioperative events, making it very unlikely to identify a single cause of POCD and resulting in the challenge of its diagnosis. Preoperative assessment of major vital organs, such as the cardiovascular and pulmonary systems, has been a routine part of preoperative assessment of the surgical patients. However, cognitive function assessment is typically not formally performed in the perioperative setting due to lack of structured screening tools and time constraint. As increasing evidence points to the fact that preexisting cognitive impairment being a risk factor for POCD and a predictor of postoperative outcomes, it seems that preoperative cognitive screening of the geriatric and even general surgical population might offer potential benefits. Other potential predisposing risk factors in addition to preexisting cognitive impairment for postoperative delirium and cognitive dysfunction are as follows^[6^–^9]^:

- Advanced age

- Poor education

- History of delirium or stroke

- Multiple comorbidities and severity of illness

- Abnormal BMI

- Functional impairment

- Duration and type of surgery (cardiac, vascular and orthopedics)

One approach is to identify those at risk of postoperative cognitive dysfunction in the preoperative setting via cognitive screen tests. One example of such screen tools is the Mini-Cog exam, which is a structured, brief and validated cognitive screening tool that can be utilized in the preoperative setting to help screen those who are at increased risk of POCD. It involves a three-item recall test for memory and a clock drawing test that serves in part as a distractor; it tests visuospatial representation, recall and executive function, and takes just minutes to complete^[10^–^12]^. Culley and colleagues showed that 24% of the patients screened positive for probable cognitive impairment (Mini-Cog score≤2) who were more likely to develop postoperative delirium compared to those with a Mini-Cog score>2^[1]^.

Yet the major challenge is to diagnose POCD correctly and in a timely manner in the postoperative period. There are multiple assessment tools available; one validated screening tool for delirium is the confusion assessment method adapted for the ICU (CAM-ICU). The CAM-ICU has been utilized in many ICUs to identify ICU delirium^[13]^. Outside of an ICU environment, the more widely accepted CAM was used to evaluate POCD in non-ICU settings^[14]^.

## What can we do to prevent and treat POCD?

There is no specific treatment available for POCD now. However, given the condition is concerning, especially to elderly patients, it is important that anesthesiologists and surgeons take it seriously and consider ways to reduce its incidence perioperatively. As POCD is most likely multifactorial, the approach to prevention and treatment should be multidisciplinary and include the anesthesia team, the surgical team and other consultants, such as geriatric specialist, when appropriate. One important area to focus on is properly screening the patients at risk, orienting patients and family members, and setting the proper postoperative expectations. For patients with significant risk of POCD and mild disease process, one may elect to proceed with conservative management of their disease if feasible in order to prevent POCD.

As we know, there are both modifiable and non-modifiable risk factors for POCD. Disease processes such as hypertension, obesity and diabetes mellitus are linked with cognitive decline in general population^[15]^. It is logical that the identification and optimization of these modifiable disease processes would assist in lowering the risk of POCD. Other social habits, such as chronic smoking and excessive alcohol consumption, is also strongly linked with delirium and long term cognitive impairment^[16]^. Therefore, discussion on smoking cessation and alcohol consumption reduction should be included in preoperative counseling, as it may have potential postoperative cognitive benefit. Lastly, optimization of a patient’s medication list in the preoperative setting may have positive impact on POCD, as polypharmacy in the elderly is extremely common and can also contribute to POCD^[3]^.

Interestingly, anesthesia per se has not been proven to have direct link to POCD^[17]^; however, it is still critical that anesthesiologists remain extra vigilant in maintaining hemodynamic stability, applying a balanced anesthetic, and minimizing intraoperative adverse events when providing anesthesia care to those who were at increased risk for POCD. In addition, advancement in technology has resulted in more sophisticated intraoperative monitoring equipment that may help prevent POCD. There is growing evidence that processed electroencephalogram (pEEG) monitoring reduces the incidence of POCD and delirium^[18^–^19]^. The use of near infrared spectroscopy (NIRS) in cardiac surgery to avoid low cerebral oxygen saturation might also be useful^[20^–^21]^.

In summary, as the aging population continues to increase, accompanying it will be the increasing number of elderly patients undergoing surgical interventions requiring anesthesia care. It has been well documented that age itself is the number one risk factor for POCD and the other major risk factor is preexisting cognitive dysfunction. It has also been shown that POCD can be detrimental to patient outcome from a clinical and economical perspective. Unfortunately, there is a lack of definitive treatment other than the aforementioned multimodal preventative strategies. There remains a significant need for future research to provide better screening and diagnostic tools and treatments for those patients at risk of POCD in the perioperative period. At this point, however, should we apply the current knowledge and integrate a standardized cognitive assessment tool into our daily preoperative assessments of our surgical patients? Given the fact that cognitive dysfunction is more common in elderly patients and those with baseline cognitive dysfunction, it is important to identify the high-risk patient population before surgery and address those at higher risk of POCD. The authors believe that the benefit of a simple extra assessment likely outweighs the additional time one spent to perform it.
